# Bilateral Visual Loss as Presenting Symptom of Posterior Reversible Encephalopathy Syndrome in a Patient with HIV/Tuberculosis Coinfection: A Case Report

**DOI:** 10.1155/2012/850176

**Published:** 2012-12-03

**Authors:** S. Guerriero, L, Ciracì, T. Centoducati, F. Pignatelli, V. Lamargese, A. Salvati, F. Dicuonzo

**Affiliations:** ^1^Department of Neurosciences and Sense Organs, University of Bari, Piazza Giulio Cesare 11, 70124 Bari, Italy; ^2^Department of Infectious Disease, University of Bari, Piazza Giulio Cesare 11, 70124 Bari, Italy

## Abstract

Posterior reversible encephalopathy syndrome (PRES) is a neurotoxic state accompanied by a unique brain imaging pattern. This cliniconeuroradiological entity usually presents with visual disturbances (cortical blindness, homonymous hemianopia, visual neglect, and blurred vision) along with neurotoxic manifestations. Only a few cases of PRES have previously been reported in patients with advanced HIV disease. The authors describe a case of posterior reversible encephalopathy syndrome (PRES) in a patient with advanced HIV/TBC infection who developed a neurotoxic state following TB and ART therapy initiation. They present a comprehensive review of the literature and discuss the pathogenetic hypotheses.

## 1. Introduction

Posterior reversible encephalopathy syndrome (PRES), also known as reversible posterior leukoencephalopathy syndrome (RPLS), is a syndrome characterized by headache, confusion, seizures, and visual loss.It is usually seen on computed tomographic scans as white-matter vasogenic edema predominantly affecting the posterior occipital and parietal lobes of the brain [1, 2]. Risk factors include malignant hypertension, eclampsia, medications such as immunosuppressants, chemotherapy, and biotherapy, and renal failure. 

Very few HIV-positive patients with PRES have been reported. We here present an AIDS patient with disseminated (including central nervous system) tuberculosis (TB) who developed PRES following TB and antiretroviral therapy (ART) initiation.

## 2. Case Report

In May 2010 a 30-years-old African female patient with preexisting compensated renal failure was brought to the attention of the Infectious Disease Department for high fever, headache, abdominal pain, renal failure (serum blood urea nitrogen 35 mg/dl, creatinine 9.5 mg/dl), severe anemia (Hb 5 mg/dl), metabolic acidosis (arterial blood gas analysis: ph: 7.33; pCO_2_: 28 mmHg; HCO_3_-(c): 14,8 mmol/L), and high blood pressure 180/100. She was diagnosed with advanced HIV infection (CD4 count, 29/*μ*l, HIV-1-RNA viral load >than 750 000 copies/ml) and disseminated tuberculosis (TB). (Quantiferon test was positive, and this result was confirmed by the study of the sputum which was positive for the presence of alcohol acid resistant bacilli.) Total body CT spotted the presence of an abdominal abscess, supposedly TBC related. The ophthalmological examination showed a normal fundus and a visual acuity of 20/20.

Antihypertensive therapy (Ramipril cp 5 mg 1 cp/day), three times a week, dialysis through groin venous access, and antituberculosis therapy (rifampicin cp 600 mg, 1 cp/day, isoniazid cp 200 mg, 1 cp + 1/2 cp/day, pyrazinamide cp 500 m, 3 cp/day, ethambutol cp 1000 mg, 1 cp 3/week, after dialysis and cotrimoxazole) were started. Calcium therapy was also administered occasionally after dialysis for a moderate hypocalcaemia (7.4 mg/dl, normal range 8.5–10.1 mg/dl).

In September she started antiretroviral therapy (ART): Lopinavir/Ritonavir cp 200/50 mg (2cp × 2/day) and raltegravir (Isentress) cp 400 mg (1 cp × 2/day). At this time her blood pressure was 130/80 mmHg.

**Figure 1:**
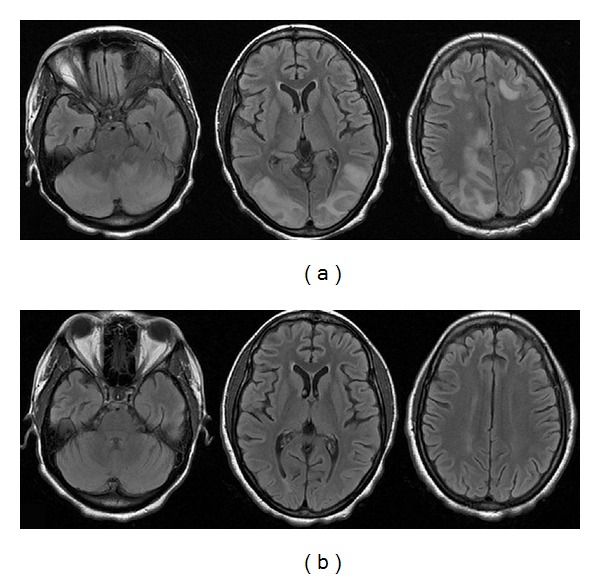
(a) Axial MRI scan (FLAIR sequence) displays multiple bilateral hyperintense lesions affecting the white matter of cerebellar hemispheres and of frontal, occipital, and parietal lobes. (b) At followup (one week later) the same sequence shows a complete resolution of all findings.

After two weeks from the beginning of ART, she complained about severe asthenia, functional inability of her lower limbs, and severe loss of her sight. Her visual acuity at this time was limited to light perception in both eyes, pupils were equal, the pupillary reflex was torpid, and fundus examination showed a normal optic head and normal vascularization. Visual-evoked potential showed an increased latency and a decreased voltage. The other cranial nerves were observed to be intact. Lumbar puncture, executed at this time, showed no evidence of active central nervous system inflammation/infection. A new magnetic resonance imaging (MRI) (Figure 1(a)) of the brain revealed an abnormal T2 hyperintensity within both cerebellar and parietooccipital and frontal lobes. The classic features of neuroimages, combined with clinical presentation of decreased vision and headache, confirmed the diagnosis of PRES.

The patient underwent supportive therapy. She had a clinical improvement within a few days; new imaging (Figure 1(b)) showed bilateral white matter lesions with decreasing size, and her visual acuity returned to 20/20 in both eyes. The patient was discharged without neurological deficits 10 days later. After three months of followup, the patient is doing well, HI-viral load is suppressed, CD4 count has increased to 180/*μ*l, and kidney function has improved substantially.

## 3. Discussion

PRES is a neurotoxic state characterized by headache, nausea and vomit, altered mental status, seizures, coma, and visual disturbance [1]. The most common visual abnormality is cortical blindness, but homonymous hemianopia, visual neglect, and blurred vision also occur. These symptoms are thought to result from cerebral edema. PRES typically develops in the setting of a significant “systemic process,” including preeclampsia [3], transplantation [4, 5], infection/sepsis/shock [6], autoimmune diseases [7, 8], cancer chemotherapy [9, 10], and hypertensive crisis complicated with seizure [11], and it was also reported in a case of multisystem mitochondrial disorder [12]. The clinical course is characterized by variability from day to day, or even hour to hour.

The mechanism behind the development of angiogenic edema and CT and MR imaging appearance of PRES is not known. Two opposite hypotheses are commonly reported: (1) severe hypertension leads to failing autoregulation, subsequent hyperperfusion, with endothelial injury/vasogenic edema; and (2) vasoconstriction and hypoperfusion lead to brain ischemia and subsequent vasogenic edema [13]. The typical localization of the edema in the parietooccipital white matter has also been poorly understood. It probably reflects the sympathetic innervations of the brain that cause a vasoconstriction, which is stronger in the anterior circulation and weaker in the posterior [14, 15].

Typical MRI findings include white matter edema in the distribution of the posterior circulation of the brain, especially in the parietooccipital region; these abnormalities are often bilateral and can involve the grey matter. The syndrome can also affect the basal ganglia, brainstem, or, as in our patient, cerebellum and the frontal lobes. These abnormalities are best depicted by MRI: punctuate or confluent areas of increased foci on proton density and T2-weighted images. Flair sequence improves the ability to detect subtle peripheral lesions [16]. 

Only a few cases of PRES have previously been reported in patients with advanced HIV disease: Giner et al. (2002) [17] reported a case secondary to indinavir-induced hypertensive crisis; Choudhary and Rose (2005) reported a case with associated mycobacteriosis and severe hypercalcemia [18]; Saeed et al. (2007) [19] reported a case in a patient with advanced HIV, disseminated blastomycosis, and hypertension; Frank et al. (1998) [20] described an HIV+ child who developed PRES with minimal elevation of blood pressure value; Ridolfo et al. (2008) [21] reported two cases; both patients had a history of ART-related metabolic body fat tissue alterations and atypical mycobacteriosis and developed a PRES syndrome secondary to a hypertensive crisis; Michailidis et al. (2005) [22] and Küpper et al. (2010) [23] reported a patient with advanced HIV infection with disseminated (including CNS) tuberculosis (TB) and preexisting compensated renal insufficiency who developed PRES following ART and TB therapy initiation. Chang et al. (2012) reported a case of PRES potentially related to AIDS and end-stage renal disease [24].

Hypertension and endothelial damage or dysfunction are the 2 main factors involved in the development of PRES, but vascular changes, disturbed vasoreactivity, and focal blood/brain barrier breakdown have been observed in HIV-infected patients [24, 25] secondary to the use of antiretroviral drugs that impair endothelial function by means of direct mechanisms and/or their effects on lipid and glucose metabolism [26, 27]. It cannot, therefore, be excluded that the endothelial dysfunction associated to a long history of HIV infection and the exposure to antiretrovirals might have played a role in predisposing our patients to PRES despite the normal blood pressure.

PRES is a rare entity and early recognition of the signs and symptoms of PRES can prevent permanent neurologic disability.

 Ophthalmologist and radiologist must be familiar with it: ophthalmologists should perform a thorough examination to evaluate other potential causes of visual abnormalities; radiologists should be able to recognize the characteristic imaging features of this reversible pathological process for proper management and to avoid unnecessary work-ups. Finally, physicians involved in caring for HIV-infected patients should be aware of this reversible but potentially life-threatening syndrome.
